# Adult William's Syndrome: The Cause of an Unusual Vasculopathy and Biliary Abnormalities

**DOI:** 10.7759/cureus.47695

**Published:** 2023-10-25

**Authors:** Philip A Craven, Victor Wycoco, David Prentice

**Affiliations:** 1 Gastroenterology and Hepatology, Royal Perth Hospital, Perth, AUS; 2 Radiology, The Neurological Intervention & Imaging Service of Western Australia (NIISWA), Perth, AUS; 3 Neurosciences, Perron Institute for Neurological and Translational Science, Nedlands, AUS

**Keywords:** fetal alcohol spectrum disorder, genetic syndromes, alagille syndrome, hepatology, william's syndrome

## Abstract

A man in his 50s was diagnosed with William’s syndrome (WS) following the investigation of severe vasculopathy and bile duct abnormalities. The vascular lesions included: right carotid artery hypoplasia, tortuous dilated left carotid artery, severe aortic hypoplasia, and pulmonary branch arterial stenoses. The bile ducts were dilated with damaged and inflamed intrahepatic ducts. The patient had been labeled with fetal alcohol syndrome as a consequence of his mother’s alcohol addiction. The etiology is thought to be the combined effects and his genetic condition and prenatal alcohol exposure.

## Introduction

Williams syndrome (WS) occurs in 1;10,000 births and is due to a deletion of 23-26 genes on the long arm of chromosome 7(7q11.23). This results from an error in the recombination of the low-copy repeats during the meiotic process in germline cells. The genes affected include elastin (ELN), LIMK1, CLIP2, GTF2IRD1, WSTF(Baz1B), GTF2I, Fzd9, and Cyln [1]. Of these, the hemizygous deletion of the ELN gene, which encodes for elastin, is the cause of the vascular lesions. The most common result of this is supravalvular aortic stenosis, which occurs in 70% of cases. The clinical phenotype of WS is distinctive facies called “elfin face” with pointy ears, bulbous nasal tip, depressed nasal bridge, long philtrum, full lips, periorbital fullness, full lips, and malocclusion; cognitive impairment with hypersocialability and cardiovascular abnormalities including supravalvular aortic stenosis, pulmonary branch arterial stenosis, and middle aortic syndrome [2]. In fetal alcohol syndrome (FAD), the facial features (smooth or absent philtrum, small canthi, small forehead, and thin upper lip) and cognitive abnormalities (attention deficit disorder, autism) are different but have been confused in the past with WS. Congenital heart disease and vasculopathy are not recognized features of FAD [3].

## Case presentation

At age 53, a man was diagnosed with extensive vasculopathy following the investigation of syncope. There were multifocal vascular changes in the aorta, cerebral, and splanchnic arteries. He had previously been followed up in the hepatology clinic for abdominal pain post-cholecystectomy and mild liver biochemical abnormalities: alanine aminotransferase (ALT) of 65 (ref < 40 U/L) and gamma-glutamyl transferase (GGT) 153 (ref < 110 U/L). There was a background of complex congenital heart disease with hypoplastic aortic arch and severe aortic stenosis diagnosed shortly after birth. A cardiothoracic surgical review was undertaken, and surgical intervention was not necessary. He was the only child of non-consanguineous parents. Delayed childhood development and distinctive dysmorphic features (depressed nasal bridge, epicanthic folds, anteverted nares, and prominent lips with a full mouth) together with heavy maternal alcohol consumption prenatally led to a diagnosis of fetal alcohol syndrome. He had an in-hospital cardiac arrest as an infant, which resulted in possible hypoxic damage. In early childhood, he spent months in the hospital following an accidental electrocution. As an adult, he has a history of hypothyroidism and gall stones and mild asthma.

In adult life, there were recurrent hospital presentations primarily with abdominal pain, culminating in a laparoscopic cholecystectomy and an acute gastric volvulus requiring open repair. His pain persisted despite surgical intervention, and a subsequent referral to the gastroenterology subspecialty was arranged.

During the same intervening period, he had several presentations to the hospital with presyncope, palpitations, and headaches, resulting in extensive investigations including an electrocardiogram (ECG), echocardiography, and ambulatory cardiac monitoring. The ECG and ambulatory ECG showed no arrhythmias. Echocardiography showed some moderate supravalvular aortic stenosis but was otherwise normal. For the workup of headaches and recurrent syncope, computed tomography angiography of the head and thorax and magnetic resonance imaging of the brain were organized. As well as extensive blood work, including electrolytes and troponin. No definitive diagnosis was made, but it was felt that his cerebral vasculopathy and supravalvular aortic stenosis contributed to cerebral hypoperfusion.

On examination, the patient was 167.1 cm tall (10th percentile) and had a head circumference of 56.5 cm (50th percentile). He has an ejection systolic murmur and well-healed chest and abdominal scars. He exhibited dysmorphic features, including a depressed nasal bridge, epicanthic folds, anteverted nares, and prominent lips with a full mouth. Radial pulse of 52 with a regular rhythm with no radial-radial delay. The carotid pulse was more prominent on the left side. Blood pressure is 120/90 bilaterally. Heart sounds were dual, with an ejection systolic murmur, loudest over the aortic region, accentuated with deep inspiration. Femoral pulses present bilaterally-nil femoral-femoral delay. Popliteal arteries and pedal pulses present bilaterally with normal capillary refill time. There were no gross or focal neurological defects identified.

Investigations

**Figure 1:**
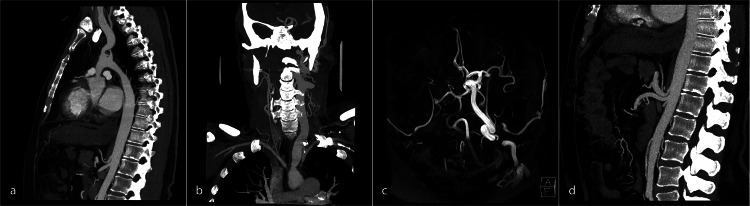
CT thoracic angiogram, head, carotid and circle of Willis angiogram.

The patient had a hypoplastic supra-valvular ascending aorta to the distal arch. The narrowing has been reported to be maximal between the left common carotid and subclavian arteries. In our patient, the aortic arch was the smallest in caliber distal to the left subclavian artery, narrowed to 12 mm over a 40 mm segment into the descending aorta (Figure 1, first panel). There were marked abnormalities in the great vessels of the aortic arch. There is a short stenosis and severe hypoplasia at the origin of the brachiocephalic artery with moderate hypoplasia of the remainder of the vessel, including the right common carotid and subclavian arteries. The right internal carotid artery (ICA) was subsequently hypoplastic (measuring 1 mm) with a congenitally small carotid canal at the skull base. The right vertebral and external carotid arteries were normal. The left common carotid was markedly abnormal and dolichoectatic, with a lumen measuring up to 15 mm (Figure 1, second panel). Intracranially, the right distal ICA and middle cerebral artery (MCA) are not visualized, with a MoyaMoya pattern of prominent collateral vessels in the right basal cistern. The left ICA and right A2 segments of the anterior cerebral artery are dolichoectatic (Figure 1, third panel). The distal aorta was small in caliber. There is a focal narrowing of the proximal celiac axis with post-stenotic dilatation (Figure 1, fourth panel). Cardiac abnormalities included dilated right coronary and left anterior descending arteries and concentric hypertrophy of the left ventricle.

**Figure 2:**
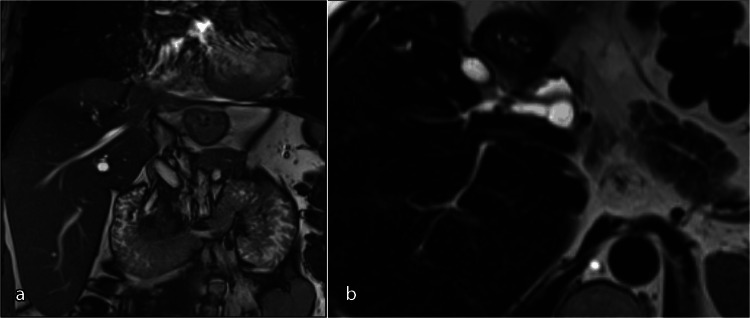
MRCP demonstrating left lobe atrophy (a) and altered right hepatic duct draining into the cystic duct (b).

Atrophy of the left lobe of the liver was subsequently demonstrated on magnetic resonance cholangiopancreatography (MRCP) with intrahepatic ductal dilatation with no intrahepatic calculi. Minor right intrahepatic duct dilatation was demonstrated with moderate extrahepatic biliary dilatation of the common hepatic and common bile ducts in the setting of a previous cholecystectomy. Other biliary anatomy was altered with the right hepatic duct draining into the cystic duct (Figure 2).

Liver biopsy

Given the mildly deranged liver function and anatomical abnormalities outlined above a liver biopsy was organized. A subsequent biopsy showed dense predominantly lymphocytic inflammation centered around the bile duct with a small number of eosinophils and plasma cells.

Genetic testing

**Figure 3:**
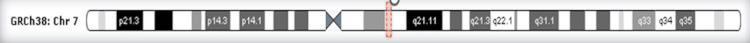
Genes deleted. The red area represents the deleted genes.

Given the vascular and biliary abnormalities, genetic testing was organized. Chromosomal microarray (CMA) testing identified a heterozygous 0.9Mb deletion on chromosome 7q11.23 (72722981_73583703). The identified deletion overlaps a contiguous gene region associated with Williams-Beuren syndrome. The deleted region includes elastin (ELN), which is considered responsible for the cardiovascular phenotype, and approximately 20 RefSeq genes (listed in the Appendix, Figure 3).

Differential diagnosis

Due to his dysmorphic features and combination of liver and cardiovascular abnormalities, a diagnosis of Alagille syndrome was suggested. Genetic testing for this was negative, and he was referred to a clinical geneticist.

Treatment and follow-up

The patient has been followed up in a range of subspecialist clinics, namely cardiology, gastroenterology/hepatology, and clinical genetics. His care to date has been fragmented by erratic attendance as well as difficulty in reaching the diagnosis, coupled with his mild cognitive impairment. Given the chronicity of the condition, no specific disease treatment has been required. The patient is being followed up in both hepatology and cardiology clinics with regard to his underlying anatomic abnormalities. With regard to his significant vascular abnormalities, cardiothoracic surgery was consulted, and no intervention was recommended.

## Discussion

Elastin role

The deletion of one copy of the elastin (ELN) gene leads to reduced elastin production and impaired quality of elastin fibers. Elastinogenesis occurs in the third trimester and extends into the neonatal period. Elastin accounts for 50% of the dry weight of large arteries and functionally allows arterial stretching in systole and recoil in diastole maintaining forward blood flow. In these arteries, it constitutes the internal elastic lamina, the concentric lamina in the media (up to 20 lamellae), and the external elastic lamina. Aside from its elastic properties, it inhibits vascular smooth muscle proliferation and binds essential extracellular matrix proteins (fibrillins and integrins) contributing to cell signaling and flow detection [4]. Decreased and defective elastin production is thought to be responsible for the vascular smooth muscle hyperplasia (lack of inhibition) and stenosis seen in WS. Elastin properties include tensile strength and durability with fibers once formed last a lifetime. The fibers are resistant to breaking down but can be degraded by metalloproteinase-forming elastokines, initiating an inflammation response with vascular wall damage. Mouse knockout models of allele elastin deletion replicate these features, and histology interestingly not only shows smooth muscle hyperplasia but also increases elastin with abnormal elastic lamellae as a compensatory mechanism. Human arterial specimens from WS patients show identical features [5].

Vascular lesions in WS

The commonest congenital lesions are supravalvular aortic stenosis (SVAS 70-75%) and branch pulmonary artery stenosis (40%) [2]. SVAS is characteristic of WS. Imaging of the large vessels reveals hypoplasia when the external lumen to internal lumen ratios are measured [4]. Stenosis occurs in other arteries, i.e., coronary, renal, mesenteric, carotid, and renal. Elastin deficiency leads to tortuous arteries, as in our patient's carotid, due to a loss of longitudinal tension [6]. Coronary lesions (20-30% cases) may be missed as they are often ostial but can be peripheral. They contribute to the increased incidence of sudden death seen in WS. Renal artery stenosis causes hypertension, accelerating vascular hypertrophy. Muscular arteries are less likely to be affected due to their reduced requirement for elastin. In a series of WS patients, no significant intracranial cerebrovascular disease was detected [7]. There have been rare cases of Moyamoya syndrome described [8], all associated with cerebral ischemic events. A careful literature search revealed no case like ours with severe carotid hypoplasia and a tortuous, severely dilated contralateral carotid. The potential pathogenesis of this will be discussed later. Our patient also had MoyaMoya syndrome-like collateralization, probably because of a unilateral hypoplastic carotid artery. Cases of Alagille [9] and PHACES syndrome [10] have been reported with carotid abnormalities similar to our patient.

A “middle aortic syndrome” [11] with narrowing of the aortic arch and descending aorta is also seen in Alagille syndrome, and neurofibromatosis type 1 is found in up to 55% of WS if appropriate imaging is performed [12]. Aortic narrowing with hypoplasia in the region of the pre- and post-subclavian arteries, as in our patient, has been described in WS [13]. The aortic narrowing in PHACES is specific to the aortic isthmus [14]. There is no increase in the frequency of aortic coarctation in infants born to mothers with the use of prenatal alcohol [15].

Neural crest in Williams and fetal alcohol syndrome

Both WS and FAD can have distinctive facial features. The face, including the skin, teeth, nerves, and blood vessels, is all derived from neural crest tissue. The cranial neural crest also provides vascular smooth muscle to the aorta, its branches, and the ductus arteriosus. In WS, a section of the deletion encompasses the WSTF gene (Williams Syndrome Transcription Factor also known as the BAZ1B bromodomain adjacent to Zinc Finger Domain 1B). Deletion of this gene causes cardiovascular abnormalities, particularly aortic hypoplasia, prominent in the section between the left carotid and the left subclavian artery. In mice models, deletion of one allele caused this abnormality in 10% of offspring [16]. Interestingly BAZ1B(WSTF) has been implicated in the facial abnormalities of WS causing a neurocristopathy [17]. This region of the genes seems to be implicated in the evolutionary self-domestication of humans by changing facial features and social interactions [18]. Alcohol is a recognized tetragen causing facial abnormalities, neurological defects (microcephaly intellectual retardation), and growth failure. As mentioned, congenital cardiac disease is uncommon but has been reported. Most of the abnormalities affect midline facial and neural structures (corpus callosum and pituitary) [3]. The effects of alcohol in embryonic development impair the neural crest by inhibiting cellular migration and adhesion, thus explaining the facial and neurological features. The neural crest is highly metabolic, and alcohol’s oxidative damage preferentially affects this structure [19]. We postulate that the combination of the genetic deletion and the alcohol use may have caused our patient's unique vascular lesions.

Bile duct abnormalities

The bile duct abnormality with the unusual vasculopathy leads us to consider Alagille syndrome (AS). Genetic testing for this was negative for JAGGED 1 (in AS 94%) and NOTCH 2 (in AS 2.5%) [20] genes. The major finding in Alagille syndrome is the paucity of intrahepatic ducts, which was not present in our patient. In WS, bile duct paucity and consequent neonatal cholestasis have been described. In our case, the liver biopsy showed bile duct inflammation and damage with no paucity of ducts. The MRI of liver and biliary tract was abnormal, with atrophy of the left lobe of liver with dilatation of the intrahepatic and extrahepatic ducts. Elastin is an important component of the submucosal space in the extrahepatic ducts contributing to elasticity and preventing bile salt damage. It is conceivable that the patient's MRI changes are a consequence of this. Biliary development is very dependent on Notch 2 signaling [20]. In Alagille syndrome, the Jagged 1 ligand is expressed on the periportal vein mesenchyme and differentiates Notch 2-expressing hepatoblasts into cholangiocytes [20]. This is what causes the duct paucity. Intrahepatic bile duct development occurs late prenatally and continues into the neonatal period. Alcohol can interfere with this pathway may have had an additive effect in our case. To our knowledge, these changes have not been reported in WS. The deletion on chromosome 7 involves genes for claudin 3 and 4. Claudin 3 is a tight junction protein that enables an epithelial barrier to toxins, especially bile and its constituents. It is conceivable that the changes seen in the liver biopsy may be a result of this, especially if bile drainage is impaired by a motility disorder.

## Conclusions

In summary, we have documented a remarkable case of Williams syndrome that emerged during our comprehensive investigation into a complex clinical presentation involving severe vasculopathy and bile duct abnormalities. The vascular anomalies encompassed right carotid artery hypoplasia, tortuously dilated left carotid artery, severe aortic hypoplasia, and pulmonary branch arterial stenoses, while the bile duct system displayed dilation with compromised and inflamed intrahepatic ducts. Intriguingly, the patient had previously been misdiagnosed with fetal alcohol syndrome due to his mother's history of alcohol addiction. Our assessment suggests that the etiology of this intricate clinical picture is likely attributable to a combination of his genetic condition and prenatal alcohol exposure. This case underscores the importance of considering a broader differential diagnosis and the need for interdisciplinary collaboration in challenging clinical scenarios.

## Appendices

ABHD11, BAZ1B, BCL7B, BUD23, CLDN3(claudin3), CLDN4(claudin4), DNAJC30, ELN (elastin), ELN-AS1 FKBP6, FZD9, LMK1, METTL27, MIR4284, MLXIPL, MIR4284, RN7SL265P, RNU61080P, RNU1198P, STX1A, TBL2, TMEM270, TRIM50, VPS37D.
